# Exercise in cancer

**DOI:** 10.4103/0971-5851.60050

**Published:** 2009

**Authors:** P. Rajarajeswaran, R. Vishnupriya

**Affiliations:** *College of Physiotherapy, Mother Theresa Post Graduate and Research Institute of Health Sciences, Puducherry – 6, India*

**Keywords:** *Exercise*, *cancer*, *prevention*, *rehabilitation*

## Abstract

Physical exercise has attracted increased interest in rehabilitation of oncological patients. The purpose of this paper is to review the literature and summarize the evidence of physical exercise in preventing cancer, its ability in attenuating the effect of cancer and its treatments and to provide guidelines for exercise prescription Review of recent literature by electronic search of MEDline (Pub Med), Cancer lit, Cochrane libraries, CINAHL were done using Keywords and the variables were identified and systematically evaluated. There is strong evidence for reduced risk of colorectal and breast cancer with possible association for prostate, endometrial and lung cancer with increasing physical activity. Exercise helps cancer survivors cope with and recover from treatment; exercise may improve the health of long term cancer survivors and extend survival. Physical exercise will benefit throughout the spectrum of cancer. However, an understanding of the amount, type and intensity of exercise needed has not been fully elucidated. There is sufficient evidence to promote exercise in cancer survivors following careful assessment and tailoring on exercise prescription.

## INTRODUCTION

More than 10 million people are diagnosed with cancer worldwide; with improvement in early detection and treatment, increasing numbers of patients can be expected to be alive five years after they are diagnosed with cancer.[1] These individuals will join the expanding number of cancer survivors, estimated at about 25 million.[1] Current cancer treatment, although increasingly efficacious for improving survival are toxic in numerous ways and produce negative short and long term physiologic and or psychological effects, including pain, decreased cardio respiratory fitness, cancer related fatigue, reduced Quality Of Life (QOL) and suppressed immune function.[2] Interest in physical activity as a means for primary prevention of cancer is increasing as the evidence for its protective effect is rapidly accumulating. The International agency for research on cancer (IARC) estimates that 25% of cancer cases worldwide are caused by overweight or obesity and a sedentary lifestyle.[3] Physical activity is an attractive cancer preventive strategy because it potentially benefits many health's end points in addition to reducing the risk of certain cancers.[4] Physical activity may have benefits throughout the spectrum of living with cancer, but cancer survivors are often at increased risk for becoming too sedentary for several reasons.

Physical exercise has attracted increased interest in rehabilitation of oncological patients in general and also in palliative care.[5] In a growing body of research that has investigated exercise in cancer patients; dramatic improvements in physiologic and psychological functioning have been documented in patients participating in exercise programs. At least 15 meta-analysis have been published reviewing 100 studies showing the nearly universal to multifactorial benefits of exercise in this patient population. Evidence of the benefits of exercise for cancer survivors in areas of psychological and quality of life (QOL) outcomes,[6] cancers related fatigue,[7] physical functioning,[8] body weight and composition,[9] muscle strength and endurance,[10] immune function[11] and cardiovascular[12–13] fitness have been reported. It may reduce the risk of cancer recurrence, second primary cancers and other chronic diseases[4,8] as well as prolong survival.[5] Exercise may also alleviate symptoms that interfere with daily life of cancer patients and survivors such as lack of appetite, diarrhea, paresthesia, constipation, physical fatigue, mental fatigue, treatment related fatigue, muscle pain, arthralgia and other pain, depression, anxiety and insomnia.[12,13,14] The purpose of this paper is to review the literature and summarize the evidence of physical exercise in preventing cancer, its ability in attenuating the effect of cancer and its treatments and to provide guidelines for exercise prescription.

## METHOD

Electronic search of MEDline (Pub Med), Cancer lit, Cochrane library, CINAHL were conducted using the following Keywords and its combinations-physical activity, exercise, prevention, intervention, cancer, neoplasm, quality of life, rehabilitation, chemotherapy, symptoms, side effects and biological mechanisms. Sources included references list of all relevant articles and reviews, Clinical Practice guidelines and Books. Review of recent systematic reviews, Meta analysis and studies on the topic that have been published in recent literature were reviewed and relevant articles are included. To be included in this review, a study had to be published in English Language between 2000 to present (Aug 09). Physical activities in cancer with multiple intervention and Pilot studies were excluded.

The following variables were identified and systematically evaluated in each paper: cancer type, age, gender, oncological treatment, QOL, biological mechanisms, type of exercise program and frequency, intensity, type and time (FITT) outcomes. Papers that met inclusion criteria and quality were studied. Definition of cancer survivor: As suggested by the national coalition of cancer survivorship to refer to any individual diagnosed with cancer from the time of discovery and for the balance of life.[15] Physical activity (PA) is defined as a bodily movement produced by skeletal muscle, which results in a substantial increase in energy expenditure over resting level. Physical exercise is defined as planned, structured, repetitive and purposeful physical activity.

## RESULTS

### Exercise in cancer prevention

Exercise may reduce the risk of developing a primary cancer, Nearly 150 studies have examined the relation between physical activity and cancer prevention at specific cancer sites, studies that meet the inclusion criteria and quality were studied and the results are summarized in Table 1 along with possible biological mechanism. The mechanism stated for association and cancer has not been established. These include changes in endogenous sexual and metabolic hormone levels,[35,36] growth factors,[60] decreased obesity and central adiposity[37] and possible changes in immune function.[65]

**Table 1:** Epidemiologic evidence on association between physical activity and cancer and possible biological mechanisms

Cancer site	Average risk reduction %	Overall level of scientific evidence	Possible mechnisms involved	Rationale
Colon[16–19]	40-50	Convincing	Decreased gastrointestinal transit time	physical activity increases gut motility and reduces mucosal exposure time to carcinogens.
			Decreased ratio of prostaglandins	Strenuous exercise may increase prostaglandin (PG) F, which inhibits colonic cell proliferation and increases gut motility while not increasing PGE2, which affects colonic cell proliferation, opposite to the effect of PGF.
			Lowered bile acid secretion or enhanced acid metabolism	Bile acid concentrations may be decreased in physically active (Confounding by diet) persons.
Breast[20–37]	30-40	Convincing	Decreased lifetime exposure to estrogen	Physical activity delays menarche, reduces the number of ovulatory cycles, and reduces ovarian estrogen production. It also reduces body fat and could reduce fat-produced estrogens. It increases the production of sex hormon-binding globulin, resulting in less biologically available estrogen.
Prostate[38–46]	10-30	Probable	Reduced exposure to testosterone	Physical activity increases production of sex hormone-binding globulin, resulting in lower levels of free testosterone
Endometrium[47,48]	30-40	Possible	Decreased percent	Fat storage of carcinogens can occur in visceral fat,
Ovary[49–51]	20-30	Insufficient	body fat	which can be released in overweight individuals.
Lung[52–53]	30-40	Possible	NE	NE
Testis[54]	10-30	Insufficient	NE	NE
All cancers[55–65]	NE	NE	Genetic predisposition of habitually active people	Constitutional factors influence athletic selection or interest in physical activity and susceptibility to cancer.
			Exercise-induced increase in antitumor immune defenses	Exercise may increase number and activity of macrophages, lymphokine-activated killer cells and their regulating cytokines; it may increase mitogen-induced lymphocyte proliferation.
			Improved antioxidant defense systems	Strenuous exercise increases the production of free radicals, whereas chronic exercise improves free radical defenses by up-regulating both the activities of free scavenger enzymes and antioxidant levels.
			Decreased circulating insulin and glucose Decreased insulin and insulin-like growth factors	Increased exercise may decrease levels of insulin and bioavailable IGF-I, both of which enhance division of normal cells and inhibit cell death.

Definitions adapted from the World Cancer Research Fund and American Institute for Cancer Research ^(1)^. Convincing evidence is defined as evidence that is conclusive; probable evidence indicates evidence is strong enough to conclude that a causal relation is likely; possible evidence indicates a causal relation may exist; insufficient evidence indicates evidence is suggestive but too sparse to make a more definitive judgment. NE not examined, IGF: Insulin-like growth factors

### Exercise benefits in cancer survivors

A majority of studies tested interventions and most of these studies used supervised exercise programs. Almost all of the studies tested aerobic exercise programs although several combined aerobic and resistance exercise programs. Most of the early studies had significant methodological limitations. Despite these limitations however, the studies have consistently demonstrated that exercise has beneficial effects on a wide variety of physical fitness and QOL endpoints in cancer survivors including functional capacity, muscular strength, body weight and composition, flexibility, fatigue, nausea, diarrhea, pain, physical well-being, functional well being, depression, anxiety, rigor, anger, mood, self esteem, satisfaction with life and overall quality of life. These studies have resulted in exercise being recommended to cancer survivors by American Cancer Society and also as a therapy for fatigue in cancer survivors.

Table 2 summarizes the recent systematic reviews of physical activity in cancer survivors

**Table 2:** Systematic reviews of physical activity in cancer survivors, published 2004-2008

Study	Studies reviewed	Authors' conclusions
Kirshbaum, 2007[66]	Systematic review of 29 intervention and observational studies in breast cancer survivors	Affirmation of the central proposition that exercise seems to be beneficial and safe for a variety of breast cancer patients continues.
Markes *et al*. 2006[67]	Systematic review and meta-analysis of 9 controlled trials in breast cancer survivors during adjuvant therapy	Improvement can be expected in physical fitness and the resulting capacity for performing activities of daily life. An improvement for other outcomes is still tenable.
McNeely *et al*. 2006[68]	Systematic review and meta-analysis of 14 randomized trials in breast cancer survivors	Exercise is an effective intervention to improve quality of life, cardio respiratory fitness, physical functioning, and fatigue.
Conn *et al*. 2006[69]	Systematic review and meta-analysis of 30 intervention studies in cancer survivors	Exercise interventions resulted in small positive effects on health and wellbeing outcomes.
Schmitz *et al*. 2005[70]	Systematic review and meta-analysis of 32 controlled trials in cancer survivors	Physical activity improves cardio respiratory fitness during and after cancer treatment, symptoms and physiologic effects during treatment, and vigor post-treatment.
Knols *et al*. 2005[71]	Systematic review of 34 controlled trials in cancer survivors	Cancer patients may benefit from physical exercise both during and after treatment.
Douglas, 2005[72]	Systematic review of 21 intervention studies in cancer survivors	There is a growing body of evidence to justify the inclusion of exercise programs in the rehabilitation of cancer patients returning to health after treatment.
Galvao and Newton, 2005[73]	Systematic review of 26 intervention studies in cancer survivors	Preliminary positive physiologic and psychological benefits from exercise when undertaken during or after traditional cancer treatment.
Stevinson *et al*. 2004[74]	Systematic review and meta-analysis of 33 controlled trials in cancer survivors	Exercise interventions for cancer patients can lead to moderate increases in physical function and are not associated with increased symptoms of fatigue.
Oldervoll *et al*. 2004[75]	Systematic review of 12 randomized controlled trials in cancer survivors	Cancer patients benefit from maintaining physical activity balanced with efficient rest periods

**Figure 1:**
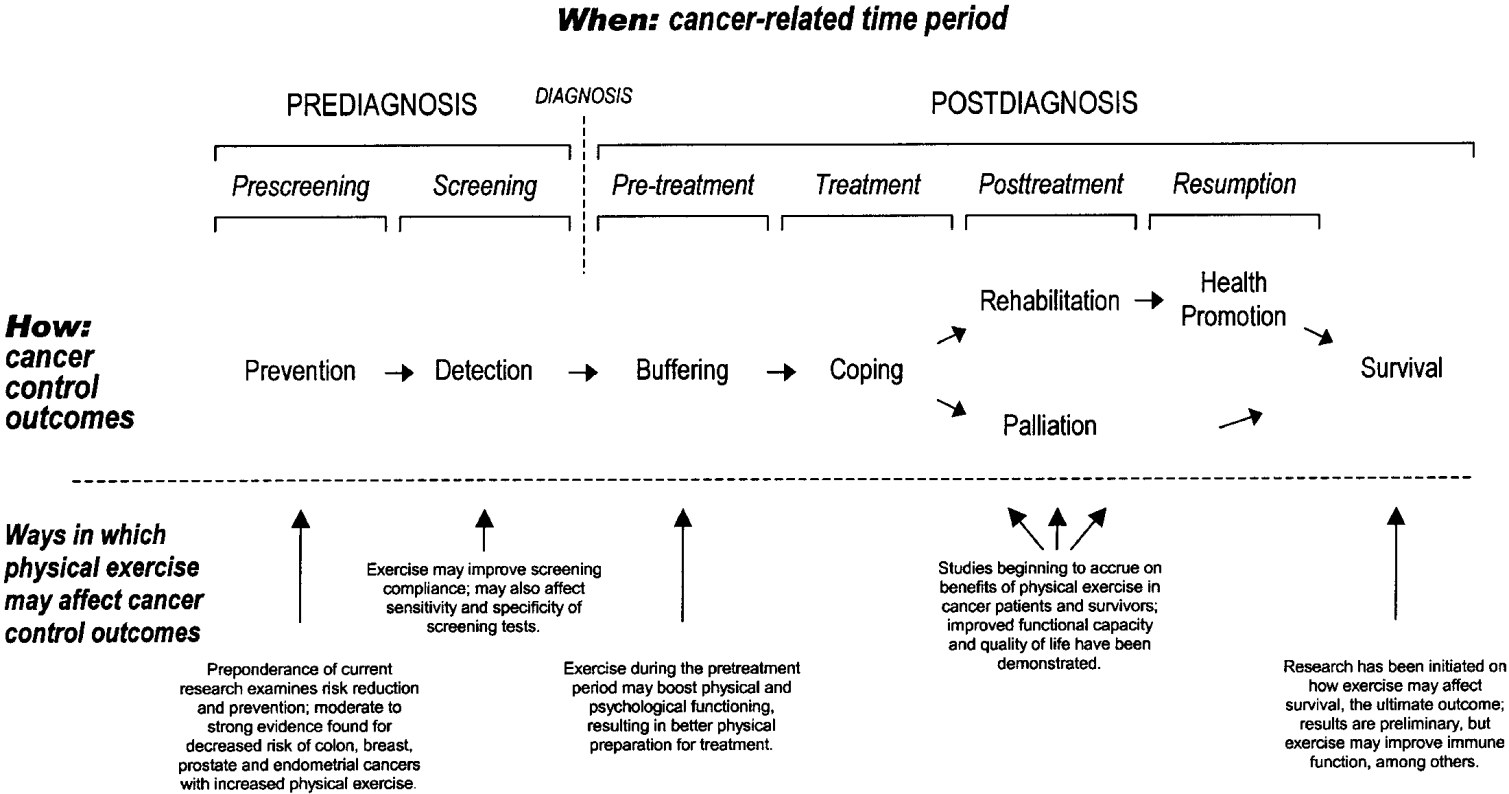
Framework PEACE: an organizational model for examining when and how physical exercise may affect the cancer experience. [Adapted from Courneya K.S, Friedenreich C.M.2]

The general consensus of these reviews is that physical activity has modest positive effect on supportive care outcomes including aerobic fitness, physical functioning, muscular strength, fatigue and some aspects of quality of life but the results are not as strong as the post-adjunct setting. In the review study by L.M. Oldervoll reported that some promising effect of physical exercises on overall Quality of life, fatigue, physical functioning, physical capacity and/or muscular fitness during and after cancer treatment.[75] C. Anderson *et al*. concluded that a six-week exercise intervention for cancer patient with or without disease and who are undergoing chemotherapy could lead to a reduction in symptoms and side effects of chemotherapy.[14] Physical activity may also help cancer survivors manage symptoms, improve mobility, slow functional decline and maintain quality of life at the end of life.[66] In a prospective phase II study, Olderwall *et al*. showed that structured physical exercise program by physiotherapist is a promising intervention for palliative cancerpatient with short life expectancy and after six weeks there was a significant decrease in physical fatigue and improvements in physical and emotional functioning and concluded that physical exercise (Resistance exercise) is a feasible intervention in a palliative care setting and may be beneficial.[76] Retraining physical function and independence in activities of daily living are important factors in palliative patient.[5,77] Figure 1 shows how and when physical exercise may affect cancer experience.

These studies suggest that physical activity may help cancer survivors live longer by: reducing the risk of cancer recurrence or slowing cancer progression and reducing the risk of other life threatening diseases including second primary cancers. The results generally show that the higher physical activity is associated with lower rate of breast and colon cancer recurrences, cancer specific mortality and all causes of mortality.

### Exercise program and prescriptions

The American College of Sports Medicine (ACSM) recommends that an exercise prescription consist of five components: frequency, intensity, time, type (FITT Principle) and progression.

Table 3 summarizes the FITT principle of exercise prescription in clinical programs.

**Table 3:** FITT exercise prescription for apparently healthy individuals

FITT principal components	Frequency	Intensity	Time	Type
Cardio respiratory (Aerobic training)	3 to 5 days per week	40% or 50-85% HRR 40% or 50-85% Vo_2_R 55% or 65-90% HRmax	20 to 60 minutes	Dynamic use of large muscle groups
Muscular strength and endurance (Resistance training)	2 to 3 days per week	12-16 RPE	1 set of 3 to 20 repetitions (e.g. 3 to 5, 8 to 10, 12 to 15)	8 to 10 exercises (All major muscles)
Flexibility	2 to 7 days per week	Stretch to tightness at the end of the range of motion but not to pain	15 to 30 seconds 2 to 4 times/ stretch	Static stretches (All major muscles)

FITT = frequency, intensity, time, type; HRR = heart rate reserve; HR max = maximum heart rate; RPE = rate of perceived exertion; VO_2_R = maximum oxygen consumption reserve. Data adapted from ACSM.

These components should be used to prescribe health related exercise training program for both healthy and chronically ill population to include patients with cancer on treatment. Suggestions to prescribe aerobic exercises for patients in early stage of cancer have been published and to date no one has published guidelines for resistance or flexibility exercise protocol in patient with cancer or recovering from cancer. It is recommended that patients should undergo a symptom-limited graded exercise test, which serves as a basis for exercise prescription. Patients present physical status and the individual's current phase of treatment or recovery must also be considered.

### Type or mode of exercise

The main health related types of exercise are aerobic, resistance and flexibility. The best mode of exercise depends on the person's goals, health status, and exercise history and cancer experience. Aerobic training is defined as a method of improving cardio respiratory system (e.g. Cycling, walking)

Resistance training is defined as a method to maintain or improve muscular strength, endurance or power, which is performed against relatively high resistance and few repetitions. Resistance exercise is a potent physiological intervention to increase muscle mass and attenuate muscle wasting. Flexibility training is defined as a method of maintaining or improving length of the muscle.

Radiation and chemotherapy may cause scar formation in joint, which may result in limitation in range of motion and this limitation can be prevented and normal range of motion can be gained by flexibility training. The best mode of exercise for the patient with cancer on treatment has not been determined because of the lack of research. However, no mode of exercise has been determined to be harmful to patient with cancer on treatment either.

### Frequency

American College of Sports Medicine recommends apparently healthy individual to engage in aerobic training 3-5 days/week. In a deconditioned population, however, several shorter exercise sessions per day are generally better tolerated. It further recommends individuals to undertake two to three non-consecutive days per week of resistance training. Flexibility training ranges from two to three days /week up to five to seven days/week.[78]

### Intensity

The intensity recommended for aerobic exercise is 40-85% of maximum heart rate calculated by Karvonen method, which calculates % of heart rate reserve.

### Karvonen formula

Age predicted maximum heart rate (APMHR) = 220 – age.

Target heart rate range = [(APHMR − resting heart rate) × per cent intensity] + resting heart rate.

However, any medication that affect's heart rate, for e.g. Beta-blockers, invalidates the formula. For these patient subjective means of gauging intensity such as rate of perceived exertion (RPE) assessed by BORG Scale can be used.[79] These individuals should be motivated or advised to exercise on intensity between 12 and 16 (somewhat hard to hard) of Borg scale. For resistance exercise, 50 -70% of 1- repetition maximum in two or three sets with 8-12 repetitions per set has been shown effective. The 1- repetition maximum refers to maximum load that a person able to lift once. Lower range of intensity is recommended for older and debilitated survivors and higher range is recommended for apparently healthy survivors. Survivors who are confined to bed or who fatigue with mild exertion may not be candidates for recommended intensity aerobic training but they may benefit from low level of physical activity. These survivors require supervision in early stages of their recovery by a physiotherapist. These severely compromised survivors may benefit from range of motion exercises and gentle resistance training within their tolerance levels in early stage of rehabilitation. The low level training will allow them to gradually build up their tolerance for activity. These survivors in early stages of recovery may later progress to short bouts walking or bicycling several times per day in order to gradually build endurance and allow them to advance to moderate intensity aerobic exercise.

For persons undergoing chemotherapy or radiation treatment the goals of exercise is to maintain function and prevent loss of endurance and strength these survivors may be benefited from routine physiotherapy and occupational therapy. Brisk walking and static cycling are some safe mode of aerobic exercise. Machine resistance and or free weights are used for resistance exercise of large muscle groups of lower and upper extremities.

### Duration of exercise

American College of Sports Medicine recommends that apparently healthy cancer survivors should exercise aerobically between 20-60 minutes, lower range for less fit and old and duration increases according to fitness and age.[78] Resistance training should be less than 60 minutes for whole bodywork. Flexibility training is given for two to four repetitions with each stretch holding for about 15-30 seconds.[78]

### Exercise progression

It depends on many variables such as the person's goals, exercise tolerance and age. Things to remember about progression are that every person will adopt differently to exercise stimulus, hence the rate of adaptation dictate the rate of progression. Treatment for cancer progresses far less predictably, and often non-linearly, because of multiple factors including the treatment schedule, fluctuating blood counts and varying symptom experiences. Definitive guidelines for progression exist except for patient with early stage disease. The guidelines state 1) Frequency and duration should be increased before intensity. 2) Progression should be slower and more gradual for the deconditioned patient and those who are experiencing severe side effects of treatment.

### Contraindications to exercise

Medical screening should be conducted for all survivors prior to their participation in an exercise program. General contraindications to exercises are cardiovascular insufficiency (e.g. uncontrolled symptomatic heart failure, acute myocarditis, and recent myocardial infarction), acute infectious diseases, metabolic diseases (e.g. thyrotoxicosis, myxedema), mental or physical impairment leading to inability to exercise.[78] In addition to general contraindication certain contraindication and precautions are specific to cancer survivors they are; Exercise within two hours of chemotherapy or radiation therapy as increased circulation may increase the effects of treatment, Intravenous chemotherapy within previous 24 hours is also a contraindication for cancer survivors.[80] Survivors with anemia (Hemoglobin <8g/dl) should not exercise until anemia is improved (Hemoglobin >10g/dl),[81] Bedside exercise programs may be prescribed for these individuals with frequent and short sessions. Hematological values where Absolute Neutrophil count less then 0.5×10 ^9^μl and Platelet count less then 50x10^9^μl is contraindication for exercise as well.[81] Acute onset of nausea during exercise and vomiting within previous 24-36 hours, unusual fatigability or muscular weakness, disorientation, blurred vision, faintness, pallor, night pain or pain not associated with injury are also signs of contraindication to exercise.[80] Survivors with immunosuppressants should avoid public gyms until there white blood cell count return to safe level (>500/mm^3^),[80] bone marrow transplanted survivors should avoid exposure to public places with risk of microbial contamination for one year after transplantation. Survivors with indwelling catheter should avoid resistance exercise of muscle in the area to avoid dislodgement of catheter. Survivors with significant peripheral neuropathies should avoid exercise of the part because of weakness or loss of balance, stationary bicycle may be used in this situation.[81]

### Adherence to exercise

The studies that reported adherence to the exercise program obtained a high level of adherence and a low dropout rate.[81–86]Adherence rate after completion of cancer treatment was 95% or higher and during active therapies was between 72-86%, which is still in acceptable level. Lower adherence rate are expected during active treatment when the patients are experiencing more symptoms. Structured exercise protocol might benefit these patients with many or severe symptoms and who struggle to regain their normal function such as those unable to resume work, or who suffer from chronic fatigue and low physical function after the end of treatment. Some cancer survivors can adopt an exercise program independently, many will benefit from referral to physiotherapist who can who can give recommendation of exercise program on type, frequency, duration, and intensity based on survivors age, previous fitness level, type of cancer, stage of treatment, type of therapy, comorbid conditions. Physiotherapy is essential for survivors with injuries, pain or specific post surgical conditions such as lymphedema or amputation.

The study by Pichett M *et al*. suggests that individual who lead sedentary lifestyle may benefit from structured exercise programs that include information and support related to exercise adherence strategy.[87] Counseling patients is one such strategy that effectively increased adherence to exercise and increased physical activity in general practice.[88] Most survivors preferred that their Oncologist initiate the discussion of exercise and such discussion also appears to increase exercise level during treatment.[89] Cancer survivors have unique and varied exercise counseling and programming preference. In the study by Jones W.L (307 survivors) 98% preferred recreational exercises, 81% preferred walking, 57% preferred unsupervised exercise.[90] These preferences for individual survivors should be considered before exercise program prescription.

Providing reassurance that exercise is safe and beneficial modality may improve exercise adherence for inactive cancer survivors and exercise program prescribed should build confidence by slowly increasing the intensity. It should be noted that adherence to exercise program is necessary to obtain improved aerobic fitness. A point to be considered is transfer of local exercise training into activities of daily living for example, resistance exercise may improve muscle strength, endurance and physical functioning but it is known that without integration of functional training improved muscle strength does not result in improved functional task performance for efficient performance of activities of daily living.[91] An individual must be able to perform basic movement and also combination of these in order to accomplish more complex tasks,[92] sports may provide training in such complex tasks. Sports are often included in exercise program to facilitate integration into daily life, as it is difficult to become physically active when sedentary.[93] Enjoyment of sports has also been reported to facilitate for adaptation of an active life style.[94] Sports might also have beneficial effect on physical activity level and physical health, develop sports specific skills, provide a sense of achievement and empowerment, develop self esteem and teach self discipline.[95] Preliminary trails on lifestyle intervention (incorporating short periods of moderate activity into their daily routine) are going on for cancer survivors and these studies have shown promising effect on improving physical functioning and quality of life and increasing physical activity.[96]

The health benefits of physical activity are independent of whether the physical activity is sport, household, occupational or recreational in nature. Some of the ways to increase physical activity that can be advised are –using stairs rather than an elevator, always walking to the destination when possible, exercising with friends and family, taking a ten minutes exercise break to stretch and quick walk, walking to visit nearby friends or co workers instead of calling them over phone, planning for active vacations rather than only driving trips, using a stationary bicycle while watching TV, planning the exercise routine to gradually increase the days per week and minutes per session.

### Adverse event issues

Of the reviewed studies 14 commented on the presence or absence of adverse events during the period of intervention. In the 14 studies 12 indicated that no harm was observed as a result of exercise during or after cancer treatment. McNeely *et al*. reported that one participant complained of nausea during one session, with no further difficulties.[83] Courneya *et al*. noted that three participants developed lymphedema out of which two had undergone axillary irradiation, a strong risk factor for lymphedema.[84] The author commented that it was not clear whether the onset of lymphedema was due to exercise.

### Exercise and diet

Overweight and obesity have been associated with many types of cancer, the ideal method to limit weight gain or loss weight is to unbalance the energy equation by combination of both diet and increasing physical activity. The expert group of WHO concluded that limiting weight gain during adult life, thereby avoiding overweight and obesity reduces the risk of postmenopausal breast cancer, colon cancer, endometrial cancer, kidney (renal cell), esophagus (adenocarcinoma) and thyroid cancer.[83] The expert committee of American Cancer Society has concluded that increasing vegetable and fruits, increasing fiber, omega 3 fatty acid, soy and limiting total fat and saturated fat have possible benefit on preventing some cancer recurrence and overall survival,[97] but the information available is insufficient to conclude the benefits for some sites. Diet with this recommendation is recommended which dietitian must individualize as food intake may be compromised by the effects of disease or therapy and to achieve specific goals of individual exercise program. It should also be noted that benefits of exercise are independent of weight loss and diet.

## DISCUSSION

The study suggests that there is strong evidence for reduced risk of some cancers with increasing physical activity. The strongest evidence exists for colorectal and postmenopausal breast cancer with possible association for prostate, endometrial and lung cancer. The findings are supported by identified biological mechanisms. The field of oncology will benefit from understanding the importance of physical activity both for primary prevention as well as in helping cancer survivors cope with and recover from treatments, improve the health of long term cancer survivors and possibly even reduce the risk of recurrence and extend survival after a cancer diagnosis. However, an understanding of the amount, type, and intensity of activity needed has not been fully elucidated for primary prevention and for patients at different stages of disease progression is still lacking. There is sufficient evidence already to recommend that at least moderate intensity activity of 30 minutes/day for five days/week or more than 45-60 minutes vigorous activities for some cancer site is given. There is sufficient evidence to promote exercise in cancer survivors following careful assessment and tailoring on exercise prescription based on health status of individual. Additional studies will be needed to more firmly establish physical activity benefits to cancer survivors.
